# Do policy-makers find commissioned rapid reviews useful?

**DOI:** 10.1186/s12961-018-0293-1

**Published:** 2018-02-26

**Authors:** Gabriel Moore, Sally Redman, Sian Rudge, Abby Haynes

**Affiliations:** 1 The Sax Institute, Level 13, Building 10, 235 Jones Street Ultimo NSW 2007, PO Box K617, Haymarket, NSW 1240 Australia; 2 School of Public Health, Sydney Medical School, The University of Sydney, Edward Ford Building (A27), Sydney, NSW 2006 Australia

**Keywords:** Knowledge translation, Rapid review, Rapid synthesis, Policy-makers, Research utilisation, Health policy

## Abstract

**Background:**

Rapid reviews are increasingly used by policy agencies to access relevant research in short timeframes. Despite the growing number of programmes, little is known about how rapid reviews are used by health policy agencies. This study examined whether and how rapid reviews commissioned using a knowledge brokering programme were used by Australian policy-makers.

**Methods:**

This study used interview data to examine the use of 139 rapid reviews by health policy agencies that were commissioned between 2006 and 2015. Transcripts were coded to identify how rapid reviews were used, the type of policy processes in which they were used, what evidence of use was provided and what reasons were given when rapid reviews were not used. Fisher’s exact test was used to assess variation between types of agencies.

**Results:**

Overall, 89% of commissioned rapid reviews were used by the commissioning agencies and 338 separate instances of use were identified, namely, on average, three uses per review. Policy-makers used reviews primarily to determine the details of a policy or programme, identify priorities for future action or investment, negotiate interjurisdictional decisions, evaluate alternative solutions for a policy problem, and communicate information to stakeholders. Some variation in use was observed across agencies. Reasons for non-use were related to changes in organisational structures, resources or key personnel in the commissioning agencies, or changes in the broader political environment.

**Conclusions:**

This study found that almost all rapid reviews had been used by the agencies who commissioned them, primarily in policy and programme development, agenda-setting, and to communicate information to stakeholders. Reviews were used mostly in instrumental and conceptual ways and there was little evidence of symbolic use. Variations in use were identified across agencies. The findings suggest that commissioned rapid reviews are an effective means of providing timely relevant research for use in policy processes and that review findings may be applied in a variety of ways.

**Electronic supplementary material:**

The online version of this article (10.1186/s12961-018-0293-1) contains supplementary material, which is available to authorized users.

## Background

Rapid review programmes have been established in response to policy-makers’ need for relevant summaries of research in short timeframes [1, 2]. Rapid reviews are intended to be tailored, targeted syntheses of research that answer specific questions arising in ‘real world’ policy or programme environments [3, 4]. There is an expanding literature about how rapid reviews may be made more useful to the policy agencies that commission them, and on the relative merits and potential limitations of their methods compared to those of other types of review [5–7].

Because of the growing number of rapid review programmes [8–14], there is an increasing interest in how rapid reviews are used. To date, this interest has focused broadly on the use of research in policy processes. For example, Pelz [15], among others [16–19], characterises use as instrumental, where research is used directly to solve a specific problem; conceptual, where it contributes to knowledge or understanding about an issue; or symbolic, where research is used to support an existing policy position or to justify decisions taken. Lomas and Brown [20] highlight the ‘functional role’ of research as it contributes to agenda-setting and to developing new or reviewing existing policies. Other scholars describe the influence of institutions, interests, ideologies and ideas on the use of research in different stages of policy development [21, 22].

These typologies have been valuable in understanding the complexity of research use in policy environments. Specifically, they indicate that one kind of use may be dominant at a particular stage in the policy process [20, 21, 23], that several kinds of use may occur concurrently [16, 24], that research may be used over lengthy timeframes [17], or that it may change in response to sudden shifts in the policy or political context [23, 25]. This suggests that use is rarely straightforward and that the environments in which decisions are made are complex.

However, little is known about how policy-makers actually use rapid reviews of research in practice [26]. Moore et al.’s [4] study of the ‘intended’ use of commissioned rapid reviews found that reviews were commonly requested in response to questions arising in planned policy processes, and that policy-makers intended to use them in both instrumental and conceptual ways. Reviews were commissioned to inform the details of policies, to identify and evaluate alternative solutions to policy problems and to determine priorities for future action or investment. The study also identified variation in intended use of reviews across different types of agencies, possibly due to their different mandate and role.

This study examines the ‘actual’ use of rapid reviews generated through the Sax Institute’s Evidence Check programme. This programme provides health policy-makers with a review of evidence from research within relatively short timeframes of between a few weeks and a few months [27]. At the commencement of an Evidence Check, policy-makers and a knowledge broker develop a structured review proposal that describes the policy issue or decision which the review will inform and articulates the questions and scope of the proposed review. The aim is to ensure the review provides policy-makers with information specific to their decision and context [4]. Since its launch in 2006, over 220 reviews have been commissioned by a range of agencies, including government departments, other government funded agencies and non-government organisations. More information on the Evidence Check process is provided in Appendix 2. Examples of Evidence Check rapid reviews are available on the Sax Institute website [28].

Specifically, this study aimed to identify (1) whether rapid reviews commissioned through the Evidence Check programme were used by the policy and programme agencies who commissioned them, (2) how they were used and how this accords with what has previously been reported, (3) whether use varied by type of organisation and (4) if reviews were not used, why not.

## Methods

### Definition of use

We defined ‘use’ broadly, to include all the activities undertaken by policy-makers in thinking about, communicating, making decisions or taking action in response (or partly in response) to the findings of a rapid review. All other definitions are provided in Appendix 1.

### Study sample

This study used de-identified data from standard quality assurance interviews. All rapid reviews commissioned and completed between January 2006 and June 2015 were included in the study. For each review, the lead policy-maker from the commissioning team was interviewed.

### Interview process

As part of standard follow-up and approximately 3–6 months after a rapid review had been completed, a brief, semi-structured interview was conducted. In addition, all agencies who commissioned a review were contacted on an annual basis to ask about later use of the reviews. These interviews included questions about the use of the review; specifically, respondents were asked about the purpose for which they commissioned the review, whether the review provided the needed information, how the review was disseminated, how and when it was used within and outside the agency and by whom, and whether and how it had influenced the agency’s decisions, policies or programmes.

In this follow-up process, policy-makers were contacted up to three times by phone or email and their consent to be interviewed was obtained. Interviews were generally conducted by telephone. However, where no contact was made, a single email containing the same questions was sent to the policy-maker inviting their response.

Not all policy-makers could be located and where the Evidence Check team felt it inappropriate (for example, the policy circumstances were politically sensitive), it did not approach the agency for an interview, although this was rare. Where the lead policy-maker no longer worked at the commissioning agency, a second team member was approached. On some occasions, it was not possible to do the follow-up interview because the staff member had left and the agency was not able to nominate an alternative team member.

Responses to questions from all interviews were transcribed from hand written notes or audiotapes and linked to the rapid review to which they pertained.

### Additional details about use

Further details about the use of reviews were separately obtained by one author (GM). Information provided in the transcripts (such as review names, publications and presentations) was used to search agency websites, online repositories and databases (such as Google Scholar, PubMed and SlideShare).

### Coding and analysis of transcripts

We developed a coding schedule to enable descriptive analyses of reviews and agencies and to categorise use, policy processes, evidence of use and reasons for non-use. In devising the schedule, we drew on the work of several authors. Our approach to categorising research use was informed by the work of Amara et al. [16], De Goede et al. [18] and Moore et al. [4]; for reasons for non-use we looked to Oliver et al. [29], Ettelt and Mays [30], and Campbell et al. [31]; and to categorise policy processes we referred to Lomas and Brown [20], Campbell et al. [31] and Flitcroft et al. [25]. For evidence of use we drew on Lavis et al.’s work [21]. During the first phase of analysis we also drew inductively on the ways in which policy-makers described use in their interviews.

We categorised types of agencies into frontline government agencies, central government, government funded and non-government organisations to identify any variation in use of reviews. Definitions of key terms are provided in Appendix 1.

The coding schedule was piloted by GM and a research assistant with experience in working with policy-makers coded all interview data. A sample of 15% of interviews was randomly selected and separately coded by GM at fortnightly intervals to examine any drift in coding, achieving an inter-rater agreement in the sample of 19 interviews of 92%. Differences were resolved in discussion.

**Fig. 1:**
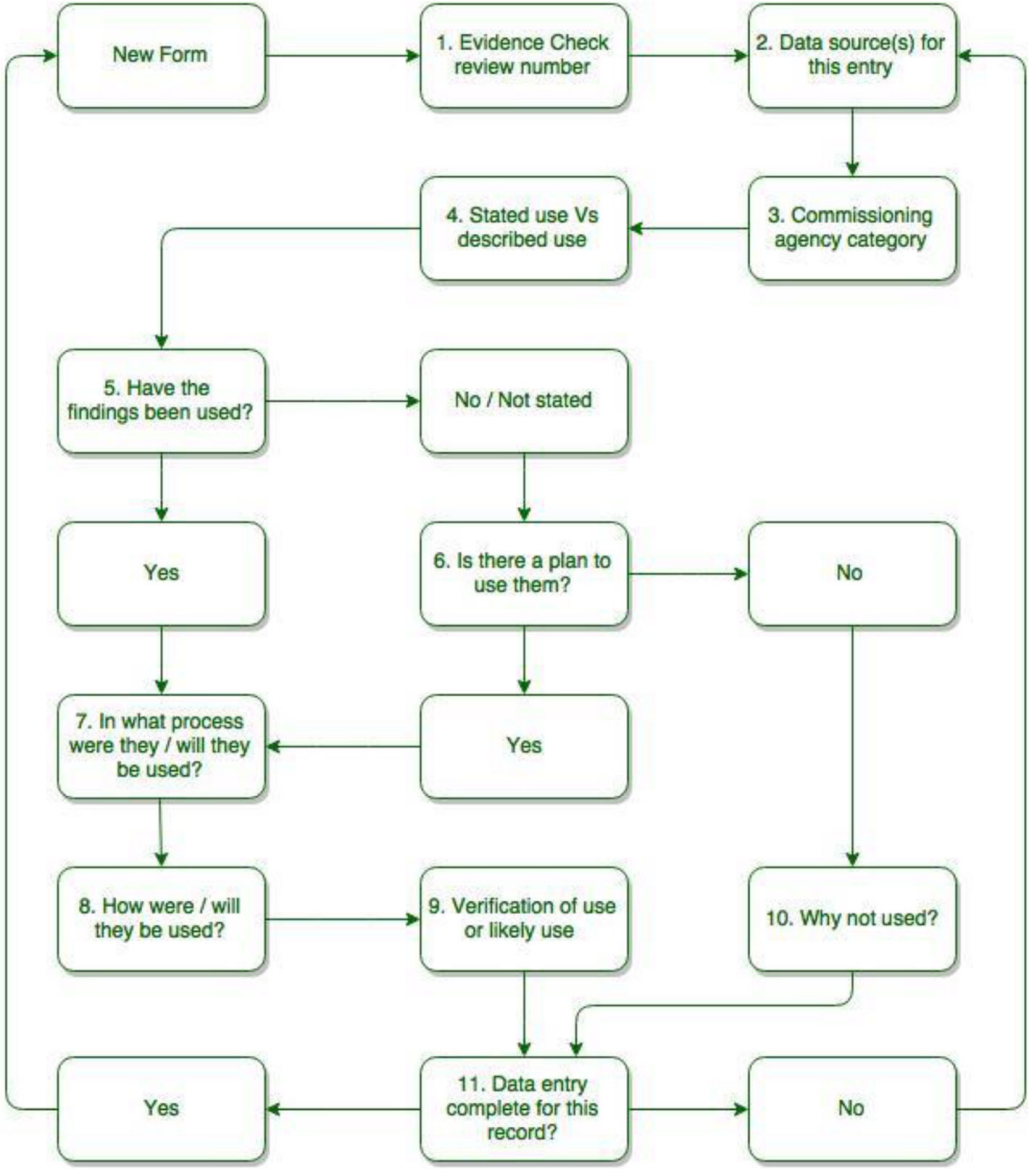
Data input and analysis

Data were entered into a form with validated fields developed in SurveyMonkey and the analysis was conducted using Excel. The data input and analysis process using SurveyMonkey is provided in Fig. 1. We compared variation between agencies using Fisher’s exact test.

## Results

### Characteristics of reviews

**Fig. 2:**
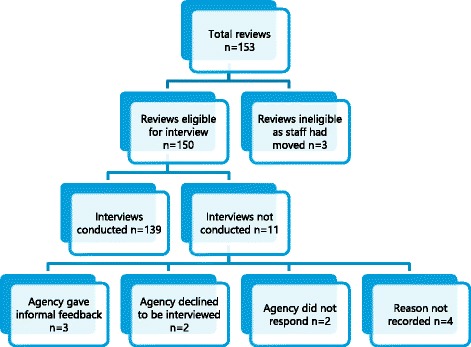
Number of eligible reviews

A total of 153 rapid reviews had been completed between the beginning of January 2006 and the end of June 2015. Three reviews were categorised as ineligible because the commissioning staff had moved and it was impossible to conduct follow-up interviews, providing a total sample of 150 reviews. Interviews were not available for 11 reviews as shown in Fig. 2.

Structured follow-up interviews had been conducted for the remaining 139 reviews (92.7% of eligible reviews). While we believe that it is unlikely that there was a systematic bias (i.e. that reviews without interviews were less likely to be used), our primary analysis was undertaken on the basis of eligible reviews. The results may therefore underestimate actual use.

Of all eligible reviews (*n* = 150), 84 reviews (56%) were commissioned by frontline government agencies, 25 (16.7%) were commissioned by central government agencies, 26 (17.3%) were commissioned by government funded agencies, 4 (2.7%) were commissioned by non-government agencies and there was no follow-up for 11 (7.3%) reviews (Table 1).

**Table 1:** Use of reviews by type of agency

	Frontline government agencies		Central government agencies		Government funded agencies		Non-government organisations		Total	
Did you use the review?	*n*	%	*n*	%	*n*	%	*n*	%	*n*	%
Yes	80	95.2	25	100	25	96.2	4	100	134	89.3
No	0	0	0	0	0	0	0	0	0	0
No, but use is planned	3	3.6	0	0	1	3.8	0	0	4	2.7
Other	0	0	0	0	0	0	0	0	0	0
Not stated	1	1.2	0	0	0	0	0	0	1	0.7
Not interviewed	n/a	0	n/a	n/a	n/a	n/a	n/a	n/a	11	7.3
Total	84	100	25	100	26	100	4	100	150	100
Was it used in one or more policy processes?										
Used in a single policy process	15	17.9	3	12	4	15.4	1	25	23	15.3
Used in multiple policy processes	68	81	22	88	22	84.6	3	75	115	76.7
Other	0	0	0	0	0	0	0	0	0	0
Not stated	1	1.2	0	0	0	0	0	0	1	0.7
Not interviewed	n/a	n/a	n/a	n/a	n/a	n/a	n/a	n/a	11	7.3
Total	84	100	25	100	26	100	4	100	150	100
In what policy process was it used?										
In policy agenda-setting	31	36.9	23	92.0	6	23.1	1	25.0	61	40.7
In research agenda-setting	1	1.2	0	0.0	2	7.7	2	50.0	5	3.3
In policy or programme development	43	51.2	2	8.0	16	61.5	1	25.0	62	41.3
In policy or programme implementation	2	2.4	0	0.0	1	3.8	0	0.0	3	2.0
In policy or programme evaluation	2	2.4	0	0.0	0	0.0	0	0.0	2	1.3
In a research process	4	4.8	0	0.0	1	3.8	0	0.0	5	3.4
Other	0	0.0	0	0.0	0	0.0	0	0.0	0	0.0
Not stated	1	1.2	0	0.0	0	0.0	0	0.0	1	0.7
Not interviewed	n/a	n/a	n/a	n/a	n/a	n/a	n/a	n/a	11	7.3
Total (all instances)	84	100.0	25	100.0	26	100.0	4	100.0	150	100.1
How was it used?										
Instrumental use										
To determine the details of a policy or programme	52	26.7	3	4.8	19	26.4	2	20	76	22.5
To identify or evaluate alternative solutions for a policy or programme	12	6.2	17	27.4	5	6.9	1	10	35	10.4
To communicate information to stakeholders or the general public	14	7.2	0	0	12	16.7	1	10	27	8
To develop a clinical guideline, protocol or resource	9	4.6	1	1.6	7	9.7	0	0	17	5
To determine the details of a research programme or process	12	6.2	0	0	2	2.8	1	10	15	4.4
To design or inform data collection, data linkage or data analysis	3	1.5	0	0	0	0	0	0	3	0.9
To determine the details of an evaluation programme or framework	1	0.5	0	0	0	0	0	0	1	0.3
Subtotal 1: Instrumental use	*103*		*21*		*45*		*5*		*174*	*51.50*
Conceptual use										
To determine priorities for future action or investment	36	18.5	18	29	11	15.3	1	11.1	66	19.5
To prepare for or negotiate a decision across agencies or jurisdictions	25	12.8	19	30.6	2	2.8	1	10	47	13.9
To understand the nature or extent of a problem	13	6.7	3	4.8	4	5.6	2	20	22	6.5
To confirm thinking or verify ideas	5	2.6	1	1.6	7	9.7	0	0	13	3.8
To create impetus for change	1	0.5	0	0	0	0	0	0	1	0.3
Subtotal 2: Conceptual use	*80*		*41*		*24*		*4*		*149*	*44.08*
Symbolic use										
To consult with stakeholders or to seek consensus	8	4.1	0	0	2	2.8	0	0	10	3
To justify or strengthen an existing policy position	4	2.1	0	0	1	1.4	0	0	5	1.5
Subtotal 3: Symbolic use	*12*		*0*		*3*		*0*		*15*	*4.44*
Subtotal 1: Instrumental use	103	52.8	21	33.9	45	62.5	5	55.6	174	51.5
Subtotal 2: Conceptual use	80	41	41	66.1	24	33.3	4	44.4	149	44.1
Subtotal 3: Symbolic use	12	6.2	0	0	3	4.2	0	0	15	4.4
Total (all instances)	195	100	62	100	72	100	9	100	338^a^	100.0
What evidence of use was provided?										
Details specified during interviews										
The ministries, agencies or people who commissioned the review	84	37.5	25	39.7	26	34.2	4	57.1	139	100.0
The policy, programme or guideline to which the findings contributed	73	32.6	23	36.5	22	28.9	2	28.6	120	86.3
The target audience(s) (if additional to those commissioning the review)	39	17.4	11	17.5	13	17.1	1	14.3	64	46.0
The forum or workshop where the findings were presented	13	5.8	1	1.6	6	7.9	0	0	20	14.4
Stakeholders groups attending the forum or workshop	10	4.5	0	0	4	5.3	0	0	14	10.1
People or agencies who requested copies of the findings	3	1.3	2	3.2	3	3.9	0	0	8	5.8
People or agencies who cited or reported the findings in a document	2	0.9	1	1.6	2	2.6	0	0	5	3.6
Other	0	0	0	0	0	0	0	0	0	0.0
Total (all instances)	224	100	63	100	76	100	7	100	370^a^	100.0
Additional details identified										
On a website, or in an online media report, or in social media	20	47.6	4	66.7	15	51.7	3	60	42	51.2
In a white paper, other consultation or discussion document	6	14.3	1	16.7	0	0	0	0	7	8.5
In a new/revised policy or programme document	4	9.5	1	16.7	2	6.9	0	0	7	8.5
In a ministerial, policy brief or summary	4	9.5	0	0	3	10.3	0	0	7	8.5
In an email, e-bulletin or newsletter	0	0	0	0	4	13.8	2	40	6	7.3
In a clinical guideline, manual or other clinical resource	4	9.5	0	0	1	3.4	0	0	5	6.1
In the agenda or records of a meeting, forum or workshop	3	7.1	0	0	1	3.4	0	0	4	4.9
In an evaluation plan, protocol or document	0	0	0	0	1	3.4	0	0	1	1.2
In a grant application, research protocol or research report	0	0	0	0	0	0	0	0	0	0.0
Other (media release)	1	2.4	0	0	2	6.9	0	0	3	3.7
Total (all instances)	42	100	6	100	29	100	5	100	82^b^	100.0
Why were reviews not used?										
The findings disagreed with an existing policy position	0	0	0	0	0	0	0	0	0	0
The findings told us nothing new about the issue	0	0	0	0	0	0	0	0	0	0
The findings were not presented in a useful way	0	0	0	0	0	0	0	0	0	0
The findings gave us insufficient information to support action	0	0	0	0	0	0	0	0	0	0
There was a change in the policy environment	2	2.4	0	0	0	0	0	0	2	1.3
There was no momentum for change in the agency or sector	1	1.2	0	0	1	3.8	0	0	2	1.3
It was difficult to integrate the findings in a policy or programme	0	0	0	0	0	0	0	0	0	0
Not stated	0	0	0	0	0	0	0	0	1	0.0
Not applicable (reviews were used)	81	96.4	25	100	25	96.2	4	100	134	90.0
Not interviewed	n/a	n/a	n/a	n/a	n/a	n/a	n/a	n/a	11	7.3
Total (all instances)	84	100	25	0	26	0	4	0	150	100
What barriers were mentioned when reviews were used?										
The findings disagreed with an existing policy position	1	1.2	0	0	0	0	0	0	1	0.7
The findings told us nothing new about the issue	0	0	0	0	1	3.8	0	0	1	0.7
The findings were not presented in a useful way	2	2.4	0	0	0	0	0	0	2	1.3
The findings gave us insufficient information to support action	0	0	0	0	0	0	0	0	0	0
There was a change in the policy environment	0	0	0	0	0	0	1	25	1	0.7
There was no momentum for change in the agency or sector	0	0	0	0	0	0	0	0	0	0.0
It was difficult to integrate the findings in a policy or programme	0	0	0	0	0	0	0	0	0	0
Other (review was not completed in a timely way)	1	1.2	0	0	0	0	0	0	1	0.7
Not stated (no barriers were mentioned)	80	95.2	25	100	25	96.2	3	75	133	88
Not interviewed	n/a	n/a	n/a	n/a	n/a	n/a	n/a	n/a	11	7.3
Total (all instances)	84	100	25	0	26	100	4	100	150	100

^a^Totals > 150: details provided about use

^b^Totals < 150: 82 uses separately identified

### Were the reviews used?

Of all eligible reviews, 134 reviews (89.3%) were used. Of the 139 reviews for which interviews were conducted, 96.4% were used. Of the five reviews where no use was reported, four policy-makers reported that they planned to use the review in the future. No information was provided about the use of one review.

From the 139 reviews for which follow-up interviews had been conducted, 338 instances of use were identified. Therefore, most reviews were used for more than one purpose.

Where use was reported, the interviewer sought further information; respondents were able to provide a reasonable level of detail about the way(s) in which the rapid review had been used (defined in Appendix 1), suggesting that respondent bias was low.

Where use was reported, interviewees were asked to describe this use in some detail. This included information about the policy-makers who used the reviews, the policies, programmes, documents and stakeholder forums in which they were used, and about the ways in which review findings were communicated to target audiences.

### How were the reviews used?

Examining all 338 instances of use, the most commonly reported uses were to determine the details of a policy or programme (22.5%, *n* = 76), to determine priorities for future action or investment (19.5%, *n* = 66), to negotiate a decision across agencies or jurisdictions (13.9%, *n* = 47), to identify or evaluate alternative actions or solutions for a policy or programme (10.4%, *n* = 35), and to communicate information to stakeholders or the general public (8.0%, *n* = 27).

The less commonly reported uses were to understand the nature or extent of a problem (6.5%, *n* = 22), develop a clinical guideline, protocol or resource (5%, *n* = 17), determine the details of a research programme (4.4%, *n* = 15), confirm thinking or verify ideas (3.8%, *n* = 13), consult with stakeholders or to seek consensus (3%, *n* = 10), justify or strengthen an existing policy position (1.5%, *n* = 5), inform data collection, data linkage or data analysis (0.9%, *n* = 3), determine the details of an evaluation programme or framework (0.3%, *n* = 1), and to provide an impetus for change (0.3%, *n* = 1). Reviews were primarily used instrumentally (51.5% of instances of use, *n* = 174) or conceptually (44.1%, *n* = 149); symbolic use was uncommon (4.4%, *n* = 15).

Examining all eligible reviews, the most commonly reported processes were policy or programme development (41.3%, *n* = 62), policy agenda-setting (40.7%, *n* = 61), policy or programme implementation (2%, *n* = 3), research agenda-setting (3.3%, *n* = 5), research development (3.4%, *n* = 5), and evaluation (1.3%, *n* = 2). This information was not provided for one review.

Further instances of the use of reviews (*n* = 82) were separately identified, following a series of online searches. Details of use were identified in websites and online reports (51%), consultation and discussion documents (9%), new or revised policies or programme documents (9%), policy briefs or summaries (9%), e-bulletins and newsletters (6%), clinical guidelines or resources (5%), meeting proceedings (4%), research reports or protocols (3%), and evaluation documents (1%; Table 1).

### Differences between frontline and central government agencies

There were some significant differences in the way reviews were used by frontline and central government agencies. Frontline government agencies were significantly more likely (26.7%, *P* = 0.002) than central government agencies (4.8%) to use reviews to determine the details of policies or programmes and significantly more likely (7.2%, *P* = 0.05) than central government agencies (0%) to use the reviews to communicate information to stakeholders or the general public.

However, central government agencies were significantly more likely (30.6%, *P* = 0.02) than frontline government agencies (12.8%) to negotiate a decision across agencies or jurisdictions and were very significantly more likely (27.4%, *P* = 0.0003) than frontline government agencies (6.2%) to identify or evaluate alternative actions or solutions for policies or programmes. No other significant differences were observed in the types of policy processes in which reviews were used.

Central government agencies were significantly more likely to use reviews in conceptual ways (66.1%, *P* = 0.05) compared to frontline government agencies (41%) and they were significantly more likely to use reviews in policy agenda-setting processes (92.0%, *P* = 0.01) than frontline government agencies (36.9%). In contrast, frontline government agencies (51.2%, *P* = 0.005) were significantly more likely to use reviews in policy and programme development, compared to central government agencies (8%). No other significant differences were observed in the types of policy processes in which reviews were used.

### What reasons were given when reviews were not used?

Four reviews had not yet been used, but use was planned. Reasons for non-use were that there had been a change in the policy agency’s structure, resources and/or key personnel (*n* = 2), or developments in the wider political environment (*n* = 2).

### What barriers were identified when reviews were used?

Interviewees mentioned an additional six instances where they encountered barriers or obstacles to use, including changes in the policy agency such as a restructure, or a lack of momentum for change in the agency or sector (*n* = 1). Other barriers mentioned included that the findings disagreed with an existing policy position (*n* = 1), provided no new information (*n* = 1), or the findings were not presented in a useful or timely way (*n* = 3). However, in all six cases, the review had been used.

## Discussion

This study found that the vast majority of Evidence Check rapid reviews had been used by the agencies that commissioned them. Instrumental, conceptual and symbolic use of reviews was reported and the reviews were used primarily in agenda-setting and to determine the details of policies and programmes. These findings shed light on the use of commissioned rapid reviews by policy agencies. We outline five of the key findings below.

First, there was clearly a very high use of the reviews by commissioning agencies; indeed, 89.3% of all 150 reviews had been used (or 96.4% of the 139 reviews for which we have systematic follow-up). The high proportion of use reported by respondents is consistent with the intended function of rapid reviews in providing timely, highly targeted, research syntheses for policy decision-making in short timeframes [6, 7, 16].

In the four reviews for which use was planned but had not yet occurred, respondents identified factors associated with the agencies or their environment, rather than with the review’s content. This suggests that the rapid reviews themselves were well suited to the needs of the policy-makers who requested them.

The high level of use may be a function of aspects of the Evidence Check process. At the commencement of each Evidence Check, the policy team and a knowledge broker develop the review questions and scope collaboratively. This pools the perspectives and expertise of both, increasing the likelihood that the final product is useful for the agencies who commissioned them. This complementarity is akin to Heaton’s third principle of co-production [32], where the knowledge and experience of each party is needed if the project’s goals are to be achieved. In addition, it is likely that the early engagement of the policy team in defining the questions and scope of rapid reviews and their ongoing involvement at key decision points in the conduct of the review, mean that the research team is given a well-specified brief and continues to receive guidance about the policy context and proposed use of the review, ensuring there is minimal drift in understanding from its first articulation to the final review. This is consistent with Oliver and Dickson [33], who found that contextually appropriate structures which support ongoing interaction between policy-makers and reviewers facilitate the production and use of research in rapid review programmes.

The high level of use may also reflect a culture among the agencies that commission reviews which is favourable to valuing and using research, or may reflect a higher level of skill in using research on the part of the commissioning policy-makers than is sometimes recognised [29, 34, 35]. Further, there is a cost associated with rapid reviews and the lead policy-makers contribute several hours of work in articulating their review needs, developing the proposal, providing clarification to the reviewers and commenting on the draft report. They may also be required to interpret and summarise the findings in a ministerial or executive brief, thereby providing a degree of ‘insider’ translation and promotion for the rapid review. It may be that the investment of individual policy-makers and the desire for a return on investment that these activities engender, together with a favourable research culture, address some of the known barriers to use [30, 33].

The experience of co-production – the interplay of policy and research expertise facilitated by the knowledge broker – may also bring about a new understanding of the research process for the policy-maker, the need for which has been highlighted in the literature [32, 36–38]. It is possible that participants who commission additional reviews may become increasingly skilled in commissioning reviews and integrating the findings in policy processes.

Second, the use of rapid reviews was more varied than has been previously reported; most reviews were used in more than one way. This differs from some who suggest that rapid reviews are likely to inform a single process, such as the development of new policies, or agenda-setting or evaluation [20], and agrees with those who point to the differential use of research over time [17]. In addition to using reviews to inform policy processes, policy-makers in this study reported using reviews to inform research development and research agenda-setting; these are new uses, highlighting policy agencies’ increasingly sophisticated understanding of the ways in which research can contribute to decision-making. Our experience is that reviews are generally commissioned for a specific purpose, particularly given the short timeframes, yet the findings reported in this study suggest that a single review may have other applications in addition to those for which they were commissioned.

This may be a feature of multifaceted policy processes, where, for example, the same review may identify alternative solutions to a policy problem and be used in a consensus process or in interjurisdictional decision-making. However, it may also be an attribute of rapid reviews not recognised previously; while Moat et al. [39] identified mechanisms by which context may influence the content of syntheses, here it appears that the findings themselves interact with or are brought to bear on the particular set of circumstances in which the policy problem was identified. For example, the findings may modify entrenched stakeholder positions (by providing an empirical evidence base), resolve an impasse (by identifying and evaluating options), build trust (by validating experience), or extend the reach of influence (by identifying implications for others affected by the policy decision). Thus, the circumstances themselves may be impacted by the rapid reviews or by the solutions generated. It is also possible that the findings trigger new processes such as the commissioning of research to address gaps in the evidence. While beyond the scope of this study, a better understanding of the relationship between rapid reviews and the policy context would be beneficial.

Third, in this study, the most common ways in which reviews were used were to determine the details of or evaluate alternative solutions for policies or programmes, to communicate information to stakeholders, to identify priorities for future action, to prepare to negotiate decisions, or to understand the nature or extent of the problem. These kinds of use align with instrumental and conceptual use, with a lesser emphasis on symbolic use. There may be several reasons for this. The boundaries between the three types of use may be more fluid than is generally considered; for example, reviews commissioned to support a consensus process may be at once instrumental (solving a direct problem), conceptual (diffusing new ideas) and symbolic (intended to persuade an audience in a particular direction). Further, while not all agree [18], there might also be a link between conceptual and instrumental use; for example, some authors have suggested that a high level of conceptual use of research among decision-makers may indicate a high likelihood of instrumental use [16, 18], it also makes intuitive sense that changes to thinking may translate into action further down the track. Amara et al. [16] suggest that complex decision-making contexts may benefit from the complementary perspectives of conceptual, instrumental and symbolic use.

The low incidence of symbolic use is inconsistent with other studies [16, 17]. It is possible that more nuanced categorisation may have identified additional instances of symbolic use. For example, our category ‘communicating information to stakeholders’ may have masked an intention to persuade an audience or to make a case for a particular option. Alternatively, it may indicate that commissioning a review is driven primarily by instrumental or conceptual need and, when the review is well targeted and delivered in a timely fashion, it has greater capacity than ‘found research’ to address that need.

Fourth, the complexity and fluidity of policy environments was reflected in respondents’ commentary about the obstacles they encountered in using reviews, even though we did not ask about barriers to use unless policy-makers stated they had not used their review. In particular, restructures, staff changes and shifts in political momentum were given as reasons for non-use. Yet, nearly all interviewees indicated that the reviews would be used irrespective of these barriers. We do not know whether or how the characteristics of these rapid reviews facilitated their use within complex policy processes, but this seems worth further investigation.

Fifth, there were significant differences in the ways central and frontline government agencies used reviews. This differential use could be related to the mandates of the agencies [4]; for example, central government agencies tend to have a more strategic cross-portfolio leadership role, where the careful evaluation of alternatives is imperative given the breadth of scope and large scale impact of policy decisions. Frontline government agencies’ focus, in contrast, is more on questions arising in planned policy processes and on implementing policies and programmes, and on supporting their engagement with stakeholders and the general public through clear communication.

There are several limitations to this study. It is possible that social desirability influenced respondents to report more instances of use than occurred; however, we feel that the high level of specificity given about reported use minimised this likelihood. Indeed, the evidence of use that was identified by us independently and in addition to that reported by policy-makers suggests that reported use in this study is an underestimate of actual use. Lastly, the findings refer to one rapid review programme (Evidence Check) and the results may not be generalisable to other rapid review programmes.

Taken together, these findings suggest that rapid reviews commissioned using knowledge brokers, with carefully defined review questions and scope tailored to a specific context, were used by the agencies who commissioned them. Reviews were used in multiple and diverse ways, suggesting they provided benefit beyond the original purpose for which they were commissioned. The ways in which they were used aligned most commonly with instrumental and conceptual use, with almost no symbolic use reported. Where barriers to use were identified, reviews had either been used or use was planned, suggesting that barriers did not prevent use. The relationship between rapid reviews and the policy context bears further examination.

## Conclusion

This study found that almost all rapid reviews had been used by the agencies that commissioned them, primarily in policy and programme development, agenda-setting and to communicate information to stakeholders. Reviews were used mostly in instrumental and conceptual ways and there was little evidence of symbolic use. Variations in use were identified across agencies. The findings suggest that commissioned rapid reviews are an effective means of providing timely relevant research for use in policy processes and that review findings may be applied in a variety of ways.

### Additional file

Additional file 1: Data extracted from quality assurance interviews. (XLSX 27524 kb)

## Appendix 1

### Definitions of key terms

1. **Types of agencies**
  - Frontline government agency: National or regional agencies or departments with responsibility for a single portfolio such as health, housing or transport, and which engage in planning and policy development, corporate and clinical governance, service redesign and delivery, resource distribution, performance management and workforce development
  - Central government agency: National or regional agencies or departments with multiple portfolios such as Treasury or Cabinet, and with responsibility for strategic policy and coordination level, who work with federal, state and territory governments to design and implement national reforms, and who support and monitor the delivery of major projects and initiatives across government departments and agencies
  - Government-funded agency: National or regional bodies, corporations or authorities which report to a minister or to a government department and which are independent or board-governed entities
  - Non-government agency: National or regional organisations that are not operating for the profit or gain of its individual members, whether these gains are direct or indirect
  - Other
2. **Types of policy process**
  - Policy agenda-setting: Determining priorities for action or investment, or informing thinking about or deciding on strategic policy directions (e.g. medium and long term)
  - Research agenda-setting: Deciding on priorities for research funding and investment; informing thinking about or identifying major research topics or issues, setting strategic research directions
  - Policy or programme development: Developing the content of a new policy or programme, or revising or modifying the content of an existing policy or programme
  - Policy or programme implementation: Developing an implementation plan, identifying options for implementing a policy or programme, or rolling out a programme
  - Policy or programme evaluation: Evaluating the impact of a policy or programme on a health system or population, developing an evaluation framework or measures, or assessing barriers or facilitators to evaluation
  - Research process: Developing a programme of research or a research framework, grant or protocol, or implementing or ‘doing’ research
  - Other (please specify)
3. **Ways in which findings were used**
  - Determine priorities for future action or investment, e.g. to inform thinking or prioritise action by an agency or agencies, to review options for investment or disinvestment, to set the agenda, to shape future directions
  - Understand the nature or extent of a problem, e.g. to understand the nature, cause or extent of a problem, to describe the parameters of a problem or issue, to assess the burden of an illness in a population
  - Determine the details of a policy or programme, e.g. to develop the content of a policy or programme or an implementation plan, to identify population groups or target groups, to design an intervention or model of care
  - Negotiate a decision across agencies or jurisdictions, e.g. to prepare for negotiations or to negotiate or discuss funding, resources, workforce or service arrangements with another agency, or to prepare for discussions or negotiations across agencies or jurisdictions
  - Identify or evaluate alternative actions or solutions for a policy or programme, e.g. identify options to address a problem, to evaluate or compare their effectiveness, or assess the contexts in which they are effective
  - Design, understand or inform data collection, data linkage or data analysis, e.g. to develop measures or processes for data collection or analysis, to identify how data can be used, to increase access to data, to select data, or to identify data sources
  - Communicate information to stakeholders or the general public, e.g. to describe the role of an agency or programme, to announce or disseminate or present new findings from research, to describe a service or intervention to potential users
  - Consult with stakeholders or to seek consensus, e.g. to seek input or agreement on a course of action or a policy or programme, to deliberate or decide on action by one or more agencies or stakeholder groups, or to come to a common view
  - Determine the details of an evaluation programme or framework, e.g. to identify questions for an evaluation, or to develop an evaluation process or framework
  - Determine the details of a research programme or process, e.g. to develop the content of a programme or research, or a grant application or research protocol, to contribute to the evidence base or to build on the body of knowledge
  - Develop a clinical guideline, protocol or resource, e.g. to determine the content of a clinical standard or guideline, or to design a clinical process or develop a tool for clinicians
  - Confirm thinking or verify ideas, e.g. to check whether there is evidence to support a new idea or new thinking or a new approach, or to check that a planned approach is comprehensive (that nothing major has been missed)
  - Justify or strengthen an existing policy position, e.g. to find evidence to support an existing viewpoint or position, or to demonstrate that a policy or programme is evidence based
  - Other (please specify)
4. **Categorisation of use**
  - Instrumental use: the direct use of research in concrete ways, such as when research is used to solve a specific problem or to answer a question
  - Conceptual use: the more diffuse ways in which research contributes to policy such as to increase knowledge or understanding about an issue or to influence thinking more broadly
  - Symbolic use: where research is used to support an existing policy position, or to justify inaction or decisions taken, or to make a case for a particular viewpoint or to persuade others
5. **A ‘reasonable’ amount of detail**
  - Having one or more descriptors of use which (1) were consistent with the policy-maker’s overall narrative, and (2) were provided with detailed reference to specific individuals, processes, documents and organisational teams; in our judgement, narrative coherence together with a detailed level of specificity indicated that use was likely to have occurred
6. **Evidence of use provided**
  - **The respondent specified one or more of the following details:** the name of the ministries, agencies or people who commissioned the review, the policy, programme or guideline to which the findings contributed/will contribute, the target audience(s) (if additional to ministries, agencies or people commissioning the review), the forum or workshop where the findings were presented, stakeholders groups attending the forum or workshop, the people or agencies who requested copies of the findings, the people or agencies who cited or reported the findings in a document
  - The authors used references to reviews, publications or presentations in the transcripts to search for additional evidence of use on agency websites, online repositories and databases (on a website, in an online media report, or in social media); in a white paper, other consultation or discussion document; in a new/revised policy or programme document; in a ministerial policy brief or summary; in a clinical guideline, manual or other clinical resource; in the agenda or records of a meeting, forum or workshop; in a grant application, research protocol or research report; in an evaluation plan, protocol or document; in an email, e-bulletin or newsletter; or other method
7. **Reasons for non-use of reviews**
  - The findings disagreed with an existing policy position, e.g. they were too sensitive politically, they did not agree with the agency’s viewpoint
  - The findings told us nothing new about the issue, e.g. the evidence gave us no new information
  - The findings were not presented in a way we could use them, e.g. the language was too academic or too difficult to understand, the answers were not synthesised, we needed a different kind of product
  - The findings gave insufficient information to support action or implementation, e.g. there were too many gaps in the evidence base, the information missed the mark, no contextual details were provided, or there were no recommendations
  - There was a change in the policy environment, e.g. there was a restructure, a change in priorities or resources, a delay in the policy process, key personnel changed, responsibilities changed, or agency mandate changed
  - There was no momentum for change in the agency or sector, e.g. there was no support at a senior level or no one designated to act on the findings, no stimulus to drive change
  - It was difficult to integrate the findings in a policy or programme, e.g. the commissioning team needed guidance on applying findings to a policy or programme
  - Other (please specify)
8. **Barriers mentioned when reviews were used**
  - As per item 6 above

## Evidence Check rapid review programme

An Evidence Check rapid review is a synthesis, summary and analysis of the best available research to inform policies and programmes. Each Evidence Check is specific to the commissioning policy team’s decision and context. Content areas vary and include system quality, capacity and financing, population health, chronic and complex conditions, consumers and clinical resources.

### Steps in the Evidence Check process

#### Knowledge brokering

The policy team completes a commissioning tool, describing the policy background, their review needs, timeframe and budget. Members of the policy team meet with a knowledge broker who works with them to clarify the needed information and to scope the review specifications. The knowledge broker drafts a review proposal, which is agreed with the policy team.

#### Identifying research experts

The Sax Institute circulates the opportunity to conduct the review and identifies suitable researchers. Brief CVs are forwarded to the policy team who select the researchers.

#### Contracting

Costs, timelines, reporting requirements, format and publishing arrangements are agreed with the agency and included in the contract.

#### Conduct of the review

The research team meets with the policy agency at key decision points during the conduct of the review. These generally include a meeting approximately a week after commencement to agree the search strategy, with a second meeting held on submission of the draft report.

#### Reporting format

The format of the review is broadly standardised through a template, which includes headings, sample content and minimal instructions. For example, reviewers are asked to provide an executive summary including the background and purpose of the review, the review questions, method, quality assessment and key findings.

In the main report, the full search strategy is provided in the methods section with a PRISMA diagram or similar. The grading system and rationale is provided along with a summary of the findings. Reviewers are directed to report against each of the review questions and to identify gaps in the evidence. The discussion section synthesises the findings and highlights the overall implications of the evidence across all questions.

#### Delivery and publication of the report

The draft report is provided to the policy team for comment and the researchers finalise the report. The final report is made available on the Sax Institute website with the consent of the agency.
